# Effect of alcohol in blood on neurological outcome and survival of patients with combination of polytrauma and head injury

**DOI:** 10.1186/cc14522

**Published:** 2015-03-16

**Authors:** S Pristovnik, M Strnad, V Vujanoviæ, T Pelcl, V Borovnik-Lesjak

**Affiliations:** 1 Health Center Dr. Adolf Drolc, Maribor, Slovenia; 2 Medical Faculty Maribor, Slovenia

## Introduction

The association between blood alcohol level (BAL) on mortality and neurologic outcome in patients with polytrauma and head injury is not clear and the data in the literature are sometimes conflicting. Some studies suggest a possible neuroprotective effect of alcohol and increased survival while others show the opposite. The rationale for this study was to investigate whether BAL has any impact on presentation, neurologic outcome and survival in patients with combination of polytrauma and head injury.

## Methods

This is a retrospective study of 43 polytraumatized patients with head injury who were intubated and treated by the prehospital unit and transported to the trauma center. Patients were grouped according to their BAL into BAL+ (>0.5 mg/l) and BAL- (≤0.5 mg/l). Inclusion criteria were age ≥18, Injury Severity Score ≥16 and head Abbreviated Injury Scale (AIS) ≥3. Physiological parameters and outcome with respect to survival to hospital discharge (STHD) and functional outcomes were analyzed. Severity of injuries was measured using the Trauma-Injury Severity Score (TRISS) and head injury using AIS. Functional outcome was measured using the Glasgow Outcome Scale (GOS), Cerebral Performance Category (CPC) and Glasgow Coma Scale (GCS) at discharge. Differences among groups were analyzed using Student's t test, the Mann-Whitney U test and the chi-square test.

## Results

BAL did not have a significant effect on presenting physiological parameters. There was a significant difference in the age of the groups, showing that patients in the BAL+ group were younger (32.6 vs. 56.8 years; 95% confidence level; *P *= 0.000). BAL had a lasting effect on functional measures of neurological outcome which showed better results in the BAL+ group; it had significantly better GOS (4.7 vs. 3.9; 95% confidence level; *P *= 0.027), and GCS at discharge was on the border of statistical significance (15 vs. 14; 95% confidence level; *P *= 0.05). Other variables (TRISS, AIS, STHD, CPC) did not show statistically significant differences among groups.

## Conclusion

Presence of alcohol in the blood had a positive effect on neurological outcome but there was no significant difference in survival. However, the positive results in neurologic outcome in the BAL+ group may also be due to the fact that this group was younger. The small number of patients in the study is another limiting factor of the interpretation. Therefore, the neuroprotective role of alcohol needs further clarification.

